# Development and validation of professional competency scale for military nurses: an instrument design study

**DOI:** 10.1186/s12912-022-00867-5

**Published:** 2022-04-18

**Authors:** Huijuan Ma, Xiaoli Zhu, Suofei Zhang, Jinyu Huang, Ya Lu, Yu Luo

**Affiliations:** 1 School of Nursing, Third Military Medical University/Army Medical University, Chongqing, P.R. China; 2 Institute of Military Preventive Medicine, Third Military Medical University/Army Medical University, Chongqing, P.R. China

**Keywords:** Competency, Scale, Psychometric testing, Military nurse

## Abstract

**Background:**

Military nurses are expected to be competent in providing quality nursing care in their assigned departments and meeting the medical needs of the military during deployment. Competency assessment is a key step in the development of a robust and competent nursing team. This study was aimed to develop the Professional Competency Scale for Military Nurses (PCSMN) and test its psychometric properties.

**Methods:**

An instrument development and validation study were conducted. Military nurses in military hospitals in eastern, southern, western, and northern China were recruited in this study. The study procedure comprised three main steps: item development (extensive literature review, the Delphi survey, and a pilot test), scale development (item analysis and exploratory factor analysis), and scale validation (confirmatory factor analysis and reliability test).

**Results:**

The 65-item PCSMN comprised four dimensions: clinical nursing knowledge and skills, military nursing knowledge and skills, professional ability, and comprehensive quality. The reliability and validity of the PCSMN were satisfactory, with the above four factors accounting for 66.9% of the total variance.

**Conclusion:**

The PCSMN is a good instrument for evaluating the competencies of military nurses in military hospitals. This may provide guidance for competency-based training.

**Supplementary Information:**

The online version contains supplementary material available at 10.1186/s12912-022-00867-5.

## Background

The mission of military hospitals includes three components: 1) to provide medical services for the community and nation; 2) to provide healthcare services anywhere during deployment; 3) to deliver health benefits to military personnel [1]. Nurses are the largest component of health personnel in military hospitals, and they are expected to be competent in providing quality nursing care in their assigned departments and meeting the medical needs of the military during deployment [2]. To successfully fulfill their job, they not only have to maintain clinical competency, but also sustain military nursing competency [3]. Military nurses usually shoulder the responsibility for managing and leading the team and units; therefore, they are expected to have effective leadership and management abilities [4]. Besides knowledge, skills, and abilities, the other components of competency are also required for being a competent employees, which are self-concept, value, trait and, motive [5, 6]. Being a military nurse in military hospitals is not easy, and due to the sudden notice of deployment, they need to be fully prepared and competent to provide medical care in diverse and dynamic environments [7]. Competency-based training of military nurses is essential in the development of a robust and competent nursing team; therefore, competency assessment as a key step in competency-based training should be given more attention.

Several instruments have been developed to measure self-assessed nurse competency, such as the Nurse Competency Scale [8], Self-Evaluated Core Competencies Scale [9], and Competence Inventory for Registered Nurses [10]. However, there are few instruments to measure the competency of military nurses, for which the Readiness Estimate and Deployability Index [11] is frequently used. The Readiness Estimate and Deployability Index was developed to assess deployment readiness competencies of nurses in the US military in 2001 and is composed of six dimensions: clinical nursing competency, operational nursing competency, soldier and survival skills, personal/physical/psychosocial stress, leadership and administrative support, and group integration and identification.

Disease presentations have changed considerably in recent decades, and nursing has changed accordingly. Meanwhile, military medicine has also changed with varying weapon upgrading and war pattern [12]. These changes indicate that it is necessary to develop a new instrument to measure the competencies of military nurses. Thus, this study aimed to develop and validate the Professional Competency Scale for Military Nurses (PCSMN) to address this current issue. This scale may enable researchers and nursing managers to measure the self-perceived competency of nurses in military hospitals. Furthermore, this scale may provide guidance for competency-based training.

## Methods

The study protocol was approved by the Medical Ethics Committee of Army Medical University in Chongqing, China. A determination of exemption was made by the Medical Ethics Committee. To develop and validate the PCSMN, a multi-step approach including item development, scale development, and scale validation, was utilized in this study.

### Phase 1 item development

#### Item generation

An extensive literature review was conducted to generate an item pool. Nurses in military hospitals usually not only provide care in hospitals, but also in the wilderness environment. Therefore, the literature review focused not only on the competency scale and framework of nurses, but also on the competency scale or framework of military nurses. The item pool in this study was built based on several competency scales and competency frameworks, including the Nurse Competency Scale [8], Competence Inventory for Registered Nurses [10], Readiness Estimate and Deployability Index [11], and the competency framework of military nurse managers [3]. Finally, the initial item pool was composed of 76 items in five domains: clinical nursing knowledge and skills, military nursing knowledge and skills, professional ability, management ability, and comprehensive quality.

#### Item reduction

The Delphi survey, content validity survey, and pilot tests were conducted to reduce the initial items in the item pool. First, a two-round Delphi survey was used to obtain the expert reviews of the initial items. Twenty experts in military nursing and military medicine were consulted. The item rating was based on a 5-point scale: 1 (least important) to 5 (most important). When the consensus was achieved among the panel, the data collection process was stopped. Second, a content validity survey was conducted with a panel of six experts in military nursing, who were asked to rate the relevance of each item using a 4-point Likert scale: 1 (not relevant), 2 (somewhat relevant), 3 (relevant), and 4 (very relevant). The item-level content validity index (I-CVI; the ratio of the number of experts rating 3 or 4) and the scale-level content validity index (S-CVI; the average of I-CVI scores) were calculated. Items with I-CVI of more than 0.78 were retained, and the rest were deleted [13]. Third, a pilot survey of 15 military nurses working in military hospitals was conducted to estimate the mean completion time and to examine whether items were intelligible to military nurses working in military hospitals.

### Phase 2 scale development

A formal investigation with purposive sampling was carried out to develop the PCSMN. For exploratory factor analysis, the appropriate sample size was calculated to be five participants per item of scale [14]. As for confirmatory factor analysis, the appropriate sample size was 200 or more [15]. Assuming a non-response rate of 10%, 594 military nurses in the military hospitals were recruited for this study. Among them, 20 nurses competed the same questionnaire within two weeks.

#### Item analysis

Item discrimination test and item-total correlation analysis were performed. The critical ration (CR) and item-total correlation coefficient were calculated, and CR was compared between groups based on high-low-27-percent group method [16]. The items with a CR ≥ 3.5, *P*-value of high-low-27-percent group comparison less than 0.05, and item-total correlation coefficient more than 0.40, were retained, and the others were eliminated.

#### Exploratory factor analysis

Exploratory factor analysis was performed to identify the factor structure of the PCSMN [17] using SPSS 24.0 (SPSS Inc., Chicago, IL, USA). Principal component analysis and varimax rotation were used in exploratory factor analysis. The eigenvalue of factors, the scree plot, the percentage of variance explained, and factor loading of items were combined to identify the factor structure of the PCSMN. Eigenvalues ≥ 1.0, and factor loading values ≥ 0.5, were considered as the criteria [18]. In addition, the Kaiser–Meyer–Olkin (KMO) test and Bartlett’s test of sphericity were also carried out, and a KMO coefficient more than 0.8 was considered significant.

### Phase 3 scale validation

#### Confirmatory factor analysis

Confirmatory factor analysis was performed to validate the factor structure of the PCSMN developed in exploratory factor analysis using AMOS 26.0 (IBM Corp., Armonk, NY, USA). Covariance matrices and the maximum likelihood estimations were used in the confirmatory factor analysis. The acceptable model fit was determined by the following criteria: χ^2^/df ≤ 3, RMSEA < 0.08, NFI > 0.95, IFI > 0.95, TLI > 0.95, CFI > 0.95 [19]. If necessary, item parceling would be utilized to assist confirmatory factor analysis when the model fit was not ideal [20, 21]. Items in one factor were ranked according to factor loading, and the items with the highest and lowest factor loadings were combined in turn, along with their average factor loading as a new indicator [22].

#### Convergent and discriminant validity

The average variance extracted (AVE) was calculated using the output of the confirmatory factor analysis [23]. An AVE larger than 0.50 indicates acceptable convergent validity; discriminant validity is considered acceptable when the square root of AVE is larger than the correlation values between common factors [24].

#### Tests of reliability

Internal consistency, Spearman-Brown split-half reliability, and test–retest reliability were analysed for the total sample. A Cronbach’s alpha of more than 0.8 was considered desirable. Test–retest reliability was calculated among the 20 participants who competed the same questionnaire within two weeks.

## Results

### Participant characteristics

In total, 594 questionnaires were distributed. After excluding 29 invalid questionnaires, the remaining 565 questionnaires were retained for data analysis. Among the 565 valid questionnaires, a sample of 341 was randomly chosen to carry out exploratory factor analysis, and the remainder (n = 224) were used to carry out confirmatory factor analysis. Twenty participants competed the same questionnaire within two weeks, and these data were used to carry out the test–retest reliability analysis. Details of participants’ characteristics are shown in Table 1.

**Table 1:** Participant characteristic

Characteristics	Categories	Total sample (n = 565, %)	EFA sample (n = 341, %)	CFA sample (n = 224, %)	test–retest sample (n = 20, %)
Gender	Female	550 (97.3%)	331 (97.1%)	219 (97.8%)	20 (100%)
	Male	15 (2.7%)	10 (2.9%)	5 (2.2%)	0 (0%)
Marital status	Unmarried	73 (12.9%)	48 (14.1%)	25 (11.2%)	2 (10%)
	Married	477 (84.4%)	284 (83.3%)	193 (86.2%)	18 (90%)
	Divorced	15 (2.7%)	9 (2.6%)	6 (2.7%)	0 (0%)
Professional Title	Nurse	48 (8.5%)	31 (9.1%)	17 (7.6%)	1 (5%)
	Senior nurse	225 (39.8%)	130 (38.1%)	95 (42.4%)	5 (25%)
	Nurse in charge	268 (47.4%)	165 (48.4%)	103 (46.0%)	11 (55%)
	Associate director	21 (3.7%)	14 (4.1%)	7 (3.1%)	3 (15%)
	Director of nurses	3 (0.5%)	1 (0.3%)	2 (0.9%)	0 (0%)
Age (years)	≤ 29	87 (15.4%)	56 (16.4%)	31 (13.8%)	2 (10%)
	30 ~ 39	347 (61.4%)	206 (60.4%)	141 (62.9%)	8 (40%)
	≥ 40	131 (23.2%)	79 (23.2%)	52 (23.2%)	10 (50%)
Education status	College degree or lower	123 (21.8%)	76 (22.3%)	47 (21.0%)	6 (30%)
	Bachelors	417 (73.8%)	252 (73.9%)	165 (73.7%)	14 (70%)
	Masters or higher	25 (4.4%)	13 (3.8%)	12 (5.3%)	0 (0%)
Position	Nurse	305 (54%)	178 (54.1%)	117 (52.2%)	13 (65%)
	Nurse manager	260 (46%)	153 (44.9%)	107 (47.8%)	7 (35%)
Clinical career (years)	≤ 5	74 (13.1%)	50 (14.7%)	24 (10.7%)	3 (15%)
	6 ~ 10	105 (18.6%)	66 (19.4%)	39 (17.4%)	2 (10%)
	11 ~ 15	195 (34.5%)	110 (32.3%)	85 (37.9%)	2 (10%)
	16 ~ 20	99 (17.5%)	60 (17.6%)	39 (17.4%)	4 (20%)
	≥ 21	92 (16.3%)	55 (16.1%)	37 (16.5%)	9 (45%)
Department	Medicine	178 (31.5%)	109 (32.0%)	69 (30.8%)	8 (40%)
	Surgical	166 (29.4%)	113 (33.1%)	53 (23.7%)	3 (15%)
	Others	221 (39.1%)	119 (34.9%)	102 (45.5%)	9 (45%)

*EFA* represents exploratory factor analysis, *CFA* represents confirmatory factor analysis

### Item development

Initially, there were 76 items in the initial item pool. After a two-round Delphi survey, eight items were excluded and the remaining 68 items were retained. The I-CVI of these 68 items ranged from 0.83 to 1.00, and the average of S-CVI was 0.98, which indicated that PCSMN had acceptable content validity. During the pilot survey, the feedback on the PCSMN was also good, and no further adjustment were made. Finally, the final and formal PCSMN with five dimensions and 68 items, were developed. The five dimensions were clinical nursing knowledge and skills (15 items), military nursing knowledge and skills (17 items), professional ability (12 items), management ability (8 items), and comprehensive quality (16 items).

### Exploratory factor analysis and item analysis

The results of the high-low-27-percent group method showed that the CR of 68 items ranged from 10.2 to 24.3; the *P*-value of 68 items in the high-low-27-percent group comparison was less than 0.05. The results of the item-total correlation analysis showed that the correlation coefficient of 68 items ranged from 0.53 to 0.79. The above item analysis results indicated that all the 68 items were retained and further refined in the exploratory factor analysis.

A third-round of exploratory factor analysis was conducted to identify the factor structure of the PCSMN. The results of the first-round exploratory factor analysis showed that the KMO was 0.97, and the Bartlett’s Test of Sphericity was significant (*P* < 0.01). The results of the principal component analysis showed that there were eight eigenvalues greater than one, which explained 72.1% of the variance. Combining the theory of competency proposed by Spencer and Spencer [6] and the result of the scree plot, four factors were extracted from the second-round exploratory factor analysis. The orthogonal rotation of the four-factor structure revealed a clear loading pattern, except for one item with a factor loading less than 0.5, and additional two items with cross-factor loadings greater than 0.4. Specifically, factor loadings of the item “identification and assessment techniques” on the factor “military nursing knowledge and skills” and the factor “clinical nursing knowledge and skills” were 0.374 and 0.432, respectively. The factor loadings of the item “puncture technology” on the factor “military nursing knowledge and skills” and the factor “clinical nursing knowledge and skills” were 0.523 and 0.514, respectively. The factor loadings of the item “innovation ability” on the factor “military nursing knowledge and skills” and the factor “professional ability” were 0.473 and 0.426, respectively. A third-round exploratory factor analysis was conducted after excluding these three items. The KMO and the Bartlett’s Test of Sphericity also showed that the data were suitable for principal component analysis. Four factors accounted for 66.9% of the variance, and the factor loading of each item ranged from 0.503 to 0.813 (Additional file 1).

After item analysis and exploratory factor analysis, the 68-item PCSMN was developed into a scale with 65 items, and the four factors were clinical nursing knowledge and skills (15 items), military nursing knowledge and skills (17 items), professional ability (20 items), and comprehensive quality (13 items).

### Confirmatory factor analysis, convergent validity and discriminant validity

Confirmatory factor analysis was performed to validate the factor structure obtained from exploratory factor analysis. The model fit of the PCSMN was not ideal in the first-round confirmatory factor analysis, which might be due to the portion between sample size and number of items. Therefore, item parceling was used in this study, and exploratory factor analysis was conducted among all the new indicators. The factor loadings of these new indicators in the corresponding factors ranged from 0.748 to 0.895, indicating that item parceling was appropriate for the data in this study.

**Fig. 1:**
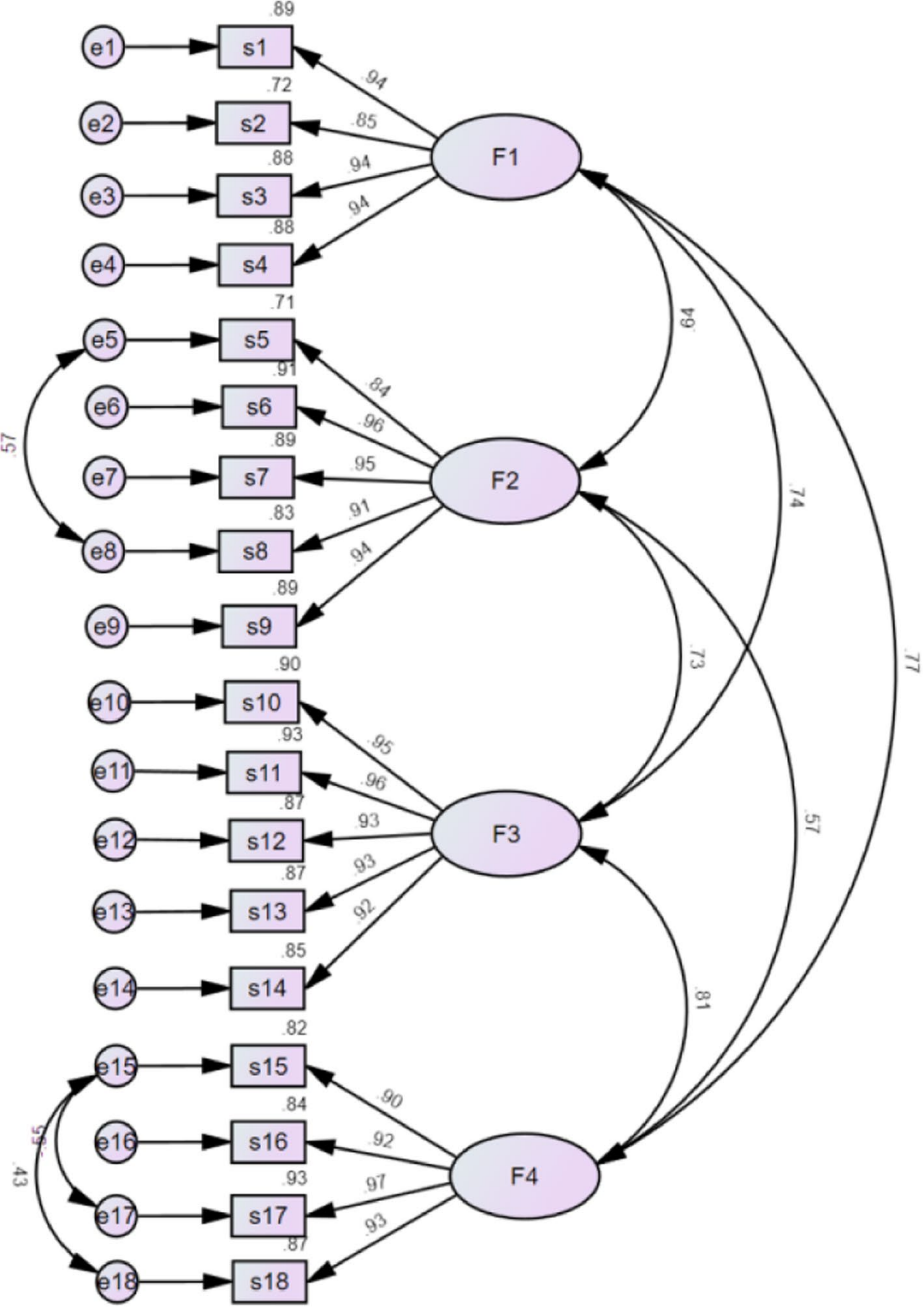
Confirmatory factor analysis of the PCSMN. The AVE ranged from 0.829 to 0.885 and CR ranged from 0.951 to 0.975, indicating that convergent validity was acceptable (Table 2) [25]. In addition, the square roots of AVE were larger than the correlation values between the common factors, indicating that discriminant validity was acceptable [24]

After item parceling, all new indicators were subjected to confirmatory factor analysis. The results showed that the partial model fit indices of the four-factor structure PCSMN were not ideal (χ^2^/df = 3.189, RMSEA = 0.099, NFI = 0.933, IFI = 0.953, TLI = 0.944, CFI = 0.953). After several modifications, the model fit of PCSMN was acceptable (χ^2^/df = 2.088, RMSEA = 0.070, NFI = 0.957, IFI = 0.977, TLI = 0.972, CFI = 0.977). In the four-factor model of the PCSMN, the standardised regression weight of all the items ranged from 0.84 to 0.97, indicating that each item could well represent the corresponding factor (Fig. 1).

### Reliability analysis

The results of the internal consistency test (Cronbach’s alpha, split-half reliability) and test–retest reliability are shown in Table 2. The coefficients of the internal consistency and test–retest reliability were greater than 0.8, indicating good internal consistency and test–retest reliability.

**Table 2:** Reliability and validity of the PCSMN

	Cronbach’s α (*n* = 565)	Split-half reliability (*n* = 565)	Test–retest reliability (*n* = 20)	AVE (*n* = 224)	CR (*n* = 224)
Clinical nursing knowledge and skills	0.954	0.906	0.940	0.829	0.951
military nursing knowledge and skills	0.968	0.932	0.974	0.853	0.967
professional ability	0.970	0.940	0.830	0.885	0.975
comprehensive quality	0.965	0.914	0.842	0.869	0.964
Total	0.983	0.889	0.922	-	-

## Discussion

This study aimed to develop and validate a competency scale for military nurses in military hospitals based on the scientific procedure for scale development. The results of the reliability and validity analysis showed that the 65-item PCSMN was a reliable and valid instrument for assessing the professional competency of military nurses in Chinese military hospitals. The PCSMN was composed of four dimensions: clinical nursing knowledge and skills, military nursing knowledge and skills, professional ability, and comprehensive quality.

The 65-item PCSMN was developed in three main steps: item development, scale development, and scale validation. During item development, an item pool of 68 items was developed through an extensive literature review, a Delphi survey, content validity surveys, and a pilot test. During scale development, three items were deleted based on the exclusion criteria of the factor analysis [25]. After analysing the factor loadings of the above three items, it was found that the item “identification and assessment techniques” and the item “puncture technology” were cross-loaded on the same two factors: “clinical nursing knowledge and skills” and “military nursing knowledge and skills”. The skills of the above two items were frequently used in both clinical and military nursing contexts, which might be the reason why these were cross-loaded. The factor loading of the item “innovation ability” was less than 0.5, which was lower than other items in the factor “professional ability”. This might be related to military values, and it is possible that the obedience and loyalty of military officers may restrict innovation to some extent [26]. During scale validation, confirmatory factor analysis showed that the four-factor structure of the 65-item PCSMN was acceptable.

It could be found that the four-factor structure of PCSMN reflects the nature of nursing competency, which is not limited to knowledge, skills, and abilities. Self-concept, value, trait, and motive, which are important job performance factors, are also indispensable for competency [5, 6]. However, these are also easily neglected in human resource management. Therefore, comprehensive quality, which includes self-concept, value, trait, and motive, is an important dimension of the PCSMN.

The “ knowledge and skills” dimension in the PCSMN is evaluated in terms of both clinical nursing and military nursing. This also reflects the nature of military nursing, as military nurses should have the competencies to provide care in both clinical and military environments [27]. The “clinical nursing knowledge and skills” dimension included knowledge and skills related to fundamental nursing, specialist nursing, regulations, and patient safety. Items in the “military nursing knowledge and skills” dimension included combat casualty care and military nursing skills. Additionally, professional ability, which comprises communication and interpersonal abilities, health education abilities, management abilities, humanistic nursing ability, research ability, and so on, is also an important dimension of the PCSMN.

Nursing competency is increasingly valued globally, and nurses are expected to possess the competencies needed to fulfill their roles [28]. Nursing competency is crucial for the quality of nursing care and the patient safety [29, 30]. Various instruments to measure self-reported competency of nurses have been used in many studies, including the Nurse Competency Scale [8], Self-Evaluated Core Competencies Scale [9], and Competence Inventory for Registered Nurses [10]. The Nurse Competency Scale is derived from Benner’s From Novice to Expert competency framework; it is a 73-item scale consisting of seven categories, including helping role, teaching-coaching, diagnostic functions, managing situations, therapeutic interventions, ensuring quality, and work role [8]. The Self-Evaluated Core Competencies Scale is designed to measure generic nursing competencies of Chinese nurses; this is a 55-item scale consisting of seven categories: clinical care, leadership, interpersonal relationship, legal/ethical practice, professional development, teaching-coaching, critical thinking/research aptitude [9]. The Competence Inventory for Registered Nurses is a measurement model for the core competencies of nursing students, and it has two dimensions, including cognitive/performance and humanity/responsibility [10]. Compared with the above three frequently used instruments, it could be found out that the PCSMN includes all the elements of competency proposed by Spencer and Spencer [6]. It also reflects different aspects of nursing competency by identifying the factor structure from the perspective of competency. Therefore, the PCSMN might have more practical value in guiding competency-building in military nurse.

In the military nursing context, the Readiness Estimate and Deployability Index is the only publicly available instrument for measuring the self-reported competency of military nurses [11]. It is a useful instrument that demonstrates validity and reliability; nevertheless, competency instruments in the military nursing context need to be re-examined in future due to the changing disease spectrum and patterns of warfare as well as the diverse needs of military nurses in different countries. Compared to the Readiness Estimate and Deployability Index, it could be found out that the PCSMN is a comprehensive tool for measuring competency. Military nurses undertake various training programmes to ensure readiness; therefore, a comprehensive and multi-layer competency instrument should offer constructive guidance for human resource management in military nursing.

This study had several strengths. First, an extensive literature review, Delphi survey, content validity survey, and pilot test were conducted to ensure the integral and accuracy of the scale. Second, the scale had acceptable construct validity with satisfactory *x*^*2*^/df, RMSEA, NFI, IFI, TLI, and CFI. Third, this scale also had acceptable reliability, convergent validity, and discriminant validity.

This study also had several limitations. First, the data collected for the study were self-reported by nurses, which may have compromised data accuracy. Second, as the study sample was recruited from China, it is possible that findings cannot be popularized to the global level. Therefore, future studies should be conducted in other regions and countries to examine the generalisability of the PCSMN.

## Conclusion

A new competency scale for military nurses was developed and validated in this study, and the reliability and validity of the PCSMN were also confirmed. The PCSMN can be used to measure competency levels of military nurses in Chinese military hospitals, and guide the implementation of competency-based training strategies to build robust and competent nursing teams. Future studies on the application of PCSMN can be carried out in other regions to further validate and adapt this scale for worldwide use.

## Supplementary Information

**Additional file 1.** Results of exploratory factor analysis and item-factor correlations (*n *= 341).

## Data Availability

The datasets used and/or analysed during the current study are available from the corresponding author on reasonable request.

## Abbreviations

PCSMN: Professional Competency Scale for Military Nurses
I-CVI: Item-Level Content Validity Index
S-CVI: Scale-Level Content Validity Index
CR: Critical Ration
KMO: Kaiser-Meyer-Olkin
