# Supplementary material for: Development and validation of professional competency scale for military nurses: an instrument design study
Source: BMC Nurs. 2022 Apr 18;21:90. doi: 10.1186/s12912-022-00867-5 (PMC9014578; doi:10.1186/s12912-022-00867-5)
Supplement: Supplementary file 1 — Additional file 1. Results of exploratory factor analysis and item-factor correlations (n = 341). [file 12912_2022_867_MOESM1_ESM.docx]

| **Additional file 1 Results of exploratory factor analysis and item-factor correlations (*n*=341)** | | |
| --- | --- | --- |
| CSMN item | Component loading | Item-factor  correlation |
| **Factor 1: Clinical nursing knowledge and skills** |  |  |
| Item 8: Specialized nursing knowledge in your unit | 0.758 | 0.788^**^ |
| Item 9: Specialized nursing skills in your unit | 0.746 | 0.798^**^ |
| Item 4: Medical equipment and emergency equipment | 0.743 | 0.835^**^ |
| Item 10: Emergency and critical care knowledge and skill in your unit | 0.736 | 0.809^**^ |
| Item 11: Knowledge of common drugs and emergency medicine in your unit | 0.730 | 0.793^**^ |
| Item 5: Observation and physical assessment | 0.723 | 0.838^**^ |
| Item 2: Fundamental nursing theoretical knowledge and skills | 0.715 | 0.793^**^ |
| Item 7: Nursing document | 0.708 | 0.783^**^ |
| Item 12: occupational safety protection regulations | 0.692 | 0.761^**^ |
| Item 6: Infection prevention and control | 0.672 | 0.804^**^ |
| Item 13: patient safety assessment and risk management | 0.665 | 0.789^**^ |
| Item 3: Fundamental nursing skills | 0.645 | 0.688^**^ |
| Item 14: work quality standard | 0.644 | 0.765^**^ |
| Item 1: nursing procedure | 0.617 | 0.749^**^ |
| Item 15: laws and regulations | 0.519 | 0.705^**^ |
| **Factor 2: Military nursing knowledge and skills** |  |  |
| Item 24: Care during evacuation and transit | 0.813 | 0.877^**^ |
| Item 22: Combat casualty care of special weapon | 0.811 | 0.812^**^ |
| Item 21: Combat casualty care in special environment | 0.810 | 0.854^**^ |
| Item 18: Different types of combat casualty care | 0.810 | 0.860^**^ |
| Item 20: Combat casualty care of shock patients | 0.807 | 0.869^**^ |
| Item 27: psychological intervention for the wounded | 0.793 | 0.867^**^ |
| Item 28: Usage of field rescue backpack | 0.792 | 0.863^**^ |
| Item 19: Different body parts of combat casualty care | 0.768 | 0.832^**^ |
| Item 23: Care of Field Internal Medicine | 0.768 | 0.836^**^ |
| Item 29: Nursing document writing during wartime | 0.760 | 0.847^**^ |
| Item 25: Nutrition support of the wounded | 0.759 | 0.861^**^ |
| Item 26: Cooperation of field operation | 0.744 | 0.801^**^ |
| Item 30: Sanitation and epidemic prevention | 0.694 | 0.818^**^ |
| Item 32: Survival in the wild | 0.661 | 0.761^**^ |
| Item 17: Field transfusion and blood transfusion | 0.644 | 0.739^**^ |
| Item 16: Fundamental combat casualty care knowledge and skills | 0.574 | 0.729^**^ |
| Item 31: Military basics | 0.503 | 0.681^**^ |
| **Factor 3: Professional ability** |  |  |
| Item 49: Ability to train others | 0.741 | 0.804^**^ |
| Item 47: Organization and coordination ability | 0.719 | 0.854^**^ |
| Item 39: Psychological nursing ability | 0.712 | 0.860^**^ |
| Item 40: Humanistic nursing ability | 0.704 | 0.828^**^ |
| Item 51: Emergency response capacity | 0.676 | 0.846^**^ |
| Item 45: Ability to formulate and execute plans | 0.664 | 0.801^**^ |
| Item 38: Health education ability | 0.664 | 0.840^**^ |
| Item 34: Clinical decision-making ability | 0.655 | 0.797^**^ |
| Item 35: Effective communication skills | 0.644 | 0.814^**^ |
| Item 36: Interpersonal skills | 0.640 | 0.808^**^ |
| Item 46: System thinking | 0.633 | 0.792^**^ |
| Item 52: Quality and safety awareness | 0.626 | 0.816^**^ |
| Item 33: Analysis and induction ability | 0.621 | 0.796^**^ |
| Item 41: Self-learning ability | 0.615 | 0.791^**^ |
| Item 50: Exemplary leading role | 0.613 | 0.771^**^ |
| Item 48: Teamwork ability | 0.613 | 0.756^*^ |
| Item 37: Teaching and training ability | 0.566 | 0.763^**^ |
| Item 42: Research ability | 0.561 | 0.734^**^ |
| Item 43: Actively participate in continuing education and training | 0.553 | 0.743^**^ |
| Item 44: Actively participate in various competitions | 0.536 | 0.713^**^ |
| **Factor 4: Comprehensive quality** |  |  |
| Item 58: Cautiousness | 0.821 | 0.875^**^ |
| Item 59: Professional etiquette and image | 0.814 | 0.867^**^ |
| Item 57: Empathy | 0.813 | 0.883^**^ |
| Item 60: Determination | 0.789 | 0.857^**^ |
| Item 56: Sense of responsibility | 0.767 | 0.873^**^ |
| Item 61: Self-control | 0.750 | 0.865^**^ |
| Item 63: Adaptability | 0.741 | 0.863^**^ |
| Item 53: Political literacy | 0.685 | 0.776^**^ |
| Item 65: Execution ability | 0.681 | 0.840^**^ |
| Item 62: Stress resistance | 0.631 | 0.800^**^ |
| Item 55: Professional identity | 0.606 | 0.800^**^ |
| Item 64: Strain capacity | 0.597 | 0.790^**^ |
| Item 54: Physical literacy | 0.595 | 0.759^**^ |
